# 3T Magnetic resonance imaging and computed tomography of the bovine carpus

**DOI:** 10.1186/s12917-022-03346-w

**Published:** 2022-06-22

**Authors:** Usama Hagag, Ayman El Nahas, Zakriya Ali Almohamad, Walter Brehm, Kerstin Gerlach

**Affiliations:** 1 Department of Surgery, Anesthesiology and Radiology, Faculty of Veterinary Medicine, Beni-Suef University, Beni-Suef, 62511 Egypt; 2 Department of Clinical Sciences, College of Veterinary Medicine, King Faisal University, PO Box 400, 31982 Al-Ahasa, Saudi Arabia; 3 Department for Horses, Faculty of Veterinary Medicine, University of Leipzig, An den Tierkliniken 21, 04103 Leipzig, Germany

**Keywords:** 3 Tesla, Magnetic resonance imaging, Computed tomography, Carpus, Cattle

## Abstract

**Background:**

Lameness in cattle is a major health problem and causes great economic losses. Carpal injury is a common cause of forelimb lameness in cattle. Radiography and/or ultrasonography of the carpus is a challenge due to complex anatomy of the joint. Additional imaging using computed tomography (CT) or magnetic resonance imaging (MRI) may be indispensable for reaching a decisive diagnosis. Precise evaluation of the clinical CT and MRI images necessitates an in-depth knowledge of the normal CT and MRI tissue variants. Therefore, our purpose was to provide a detailed description of the normal CT and MRI appearance of the osseous and soft tissue structures of twelve cadaveric bovine carpi using CT and 3 Tesla MRI. Carpi were frozen, transected in sagittal, dorsal and transverse planes then adjoined to their corresponding CT and MRI images.

**Results:**

The clinically significant articular and peri-articular structures of the bovine carpus were identified and characterized on the CT and MRI images. CT images provided a remarkable delineation of the cortical, subchondral, and cancellous bone. The high-field 3 Tesla MRI offered high definition and distinction of the delicate soft tissues of the bovine carpus.

**Conclusions:**

3 Tesla high-field MRI offers new opportunities in soft tissue tomography but cannot be compared with CT in terms of bone imaging. Clinicians have to determine whether CT, MRI or both imaging techniques are required in clinical situations.

## Background

The bovine carpus is a composite joint comprising three levels of articulations sharing a common fibrous capsule; although, each of which has its own synovial membrane [1]. The antebrachiocarpal joint is proximal, the middle carpal joint lies in the middle and the carpometacarpal joint is distal. The antebrachiocarpal joint is formed by the antebrachial bones and the proximal row of carpal bones. The middle carpal articulation lies between the proximal and distal carpal bones. The carpometacarpal joint is formed by the distal row of carpal bones and the proximal metacarpals [2]. The middle carpal and carpometacarpal joints are always communicated. The antebrachiocarpal and middle carpal joints are connected in 13% of cattle [3]. The bovine carpus involves six carpal bones arranged in two rows. The proximal row comprises the radial, intermediate, ulnar and accessory carpal bones. The distal row is made up of the fused second and third carpal and the fourth carpal bones [4]. The radial and intermediate carpal bones articulate proximally with the radius and distally with the fused second and third carpal bone. The ulnar carpal bone articulates proximally with radius and ulna and distally with the fourth carpal bone. The fused second and third carpal bone articulates distally with the third metacarpal bone. The fourth carpal bone articulates proximally with the intermediate and ulnar carpal bones and distally with the fourth metacarpal bone [5].

The carpal joint is referable as a common cause of forelimb lameness in cattle [6], but conclusive diagnosis through clinical and orthopedic investigations could be a challenge [7]. A presumptive diagnosis may be developed on the basis of imaging with radiography and/or ultrasonography. Radiography is the first line of investigation when bone pathology is suspected; however, it is of limited help for accurate identification of soft tissues and superimposed bony structures [8]. Ultrasonography is the most cost-effective modality for evaluation of soft tissues but it is operator dependent, restricted by bone, and attenuation of sound beams restricts its ability to explore deeper structures [9]. Indeterminate ultrasonographic/radiographic findings often require additional imaging by computed tomography (CT) and/or magnetic resonance imaging (MRI) either to integrate the ultrasonographic and/or radiographic findings or to clarify any inconclusive or equivocal finding, and thereby supporting clinical decision-making [10].

The use of CT and MRI as diagnostic tools in various fields of veterinary medicine is continuously increasing due to its wide array of applications in both companion and production animals [11]. CT and MRI allow radiologists to gauge the extent of injury, identify culprit cofactors of disease predisposition, and provide valuable preoperative information in cases that require surgical repair [12]. Effective use and interpretation of information gained by CT and MRI for diagnosis of pathological changes necessitates a thorough understanding of the normal CT and MRI imaging appearance of soft tissues and osseous structures of the region of interest [13]. To the authors’ knowledge, the CT and MRI of the bovine carpal joint have not been previously reported. Moreover, high-field 3 T MRI system has not been used in cattle yet. Therefore, the purpose of the present study was to describe the CT and high-field 3 T MRI imaging features of the carpal joint in healthy cattle and adjoin the obtained images to their corresponding gross anatomical slices in order to establish a clinically relevant reference data that would facilitate interpretation in clinical situations.

## Results

**Fig. 1:**
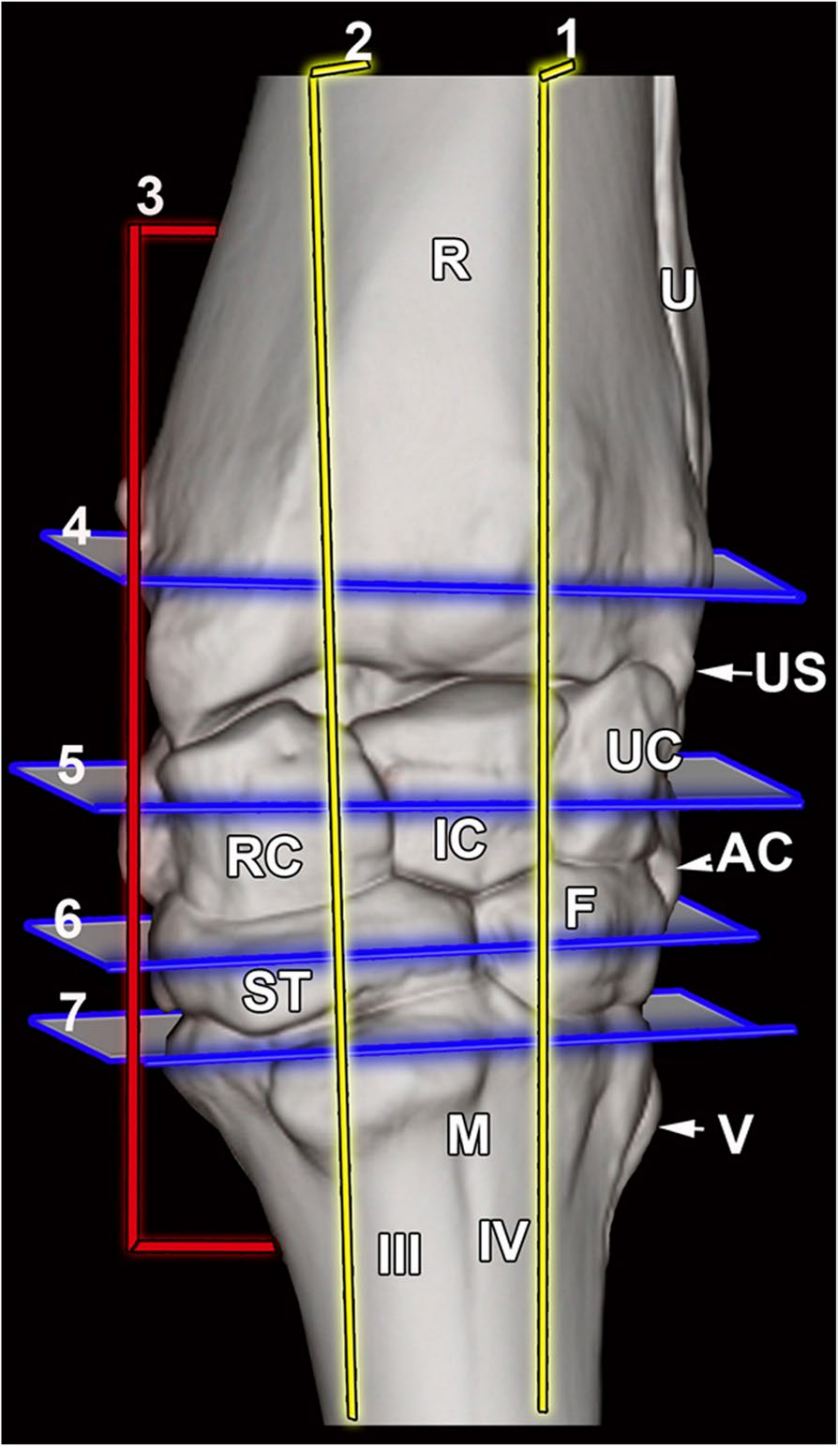
3D CT reconstructed dorsal view of the left bovine carpus showing the approximate levels of the selected CT, MRI and gross parasagittal (1–2), dorsal (3) and transverse (4–7) sections. R, radius; U, ulna; US, ulnar styloid process; UC, ulnar carpal bone; IC, intermediate carpal bone; RC, radial carpal bone; ST, fused 2nd and 3rd carpal bone; F, fourth carpal bone; AC, accessory carpal bone; M, metacarpus; III, third metacarpal bone; VI, fourth metacarpal bone; V, fifth metacarpal bone

**Fig. 2:**
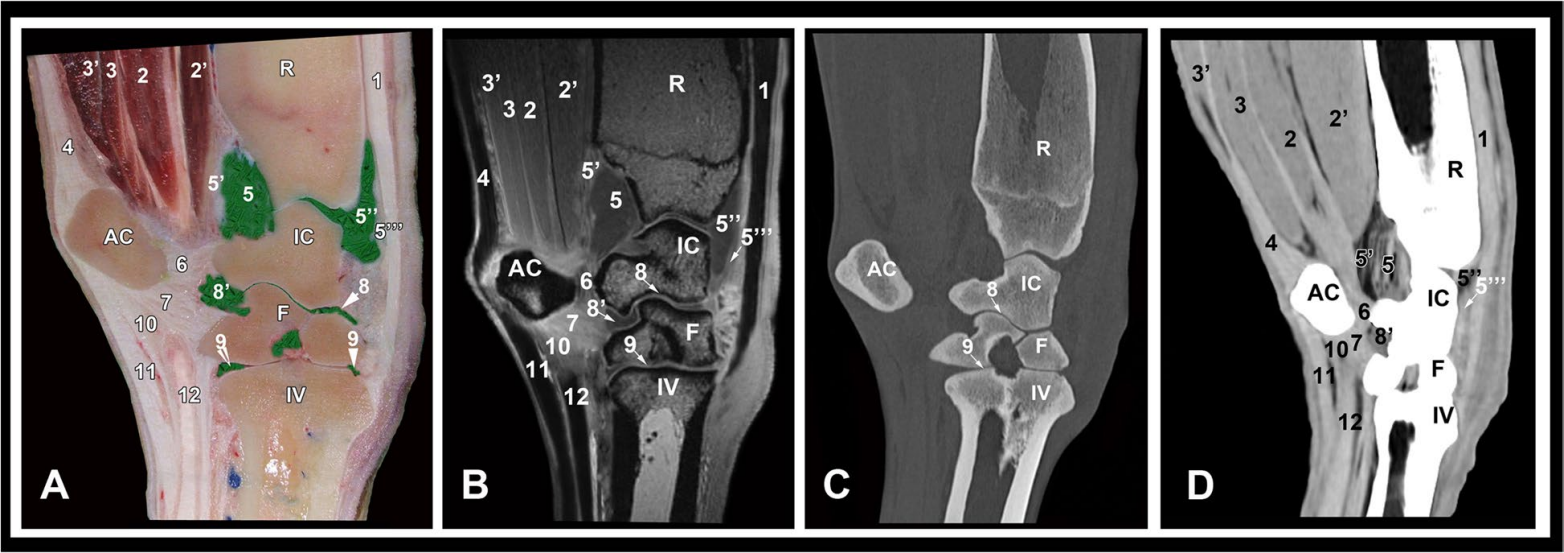
Lateral parasagittal gross anatomic **A**, MRI **B**, CT bone window **C** and CT soft tissue window (**D**) images at the level of the ulnar carpal bone. R, radius; IC, intermediate carpal bone; F, fourth carpal bone; VI, fourth metacarpal bone; AC, accessory carpal bone; 1, common digital extensor tendon; 2, ulnar and humeral heads of the deep digital flexor muscle; 2’, radial head of the deep digital flexor muscle; 3, superficial digital flexor muscle, deep part; 3’, superficial digital flexor muscle, superficial part; 4, flexor carpi ulnaris tendon; 5, palmar recess of the antebrachiocarpal joint; 5’,palmar joint capsule; 5’’, dorsal recess of the antebrachiocarpal joint; 5’’’, dorsal joint capsule; 6, accessoriocarpoulnar ligament; 7, accessorioquartal ligament; 8, middle intercarpal joint; 8’, palmar recess of the middle intercarpal joint; 9, carpometacatpal joint; 10, accessoriometacarpal ligament; 11, superficial digital flexor tendon; 12, deep digital flexor tendon

**Fig. 3:**
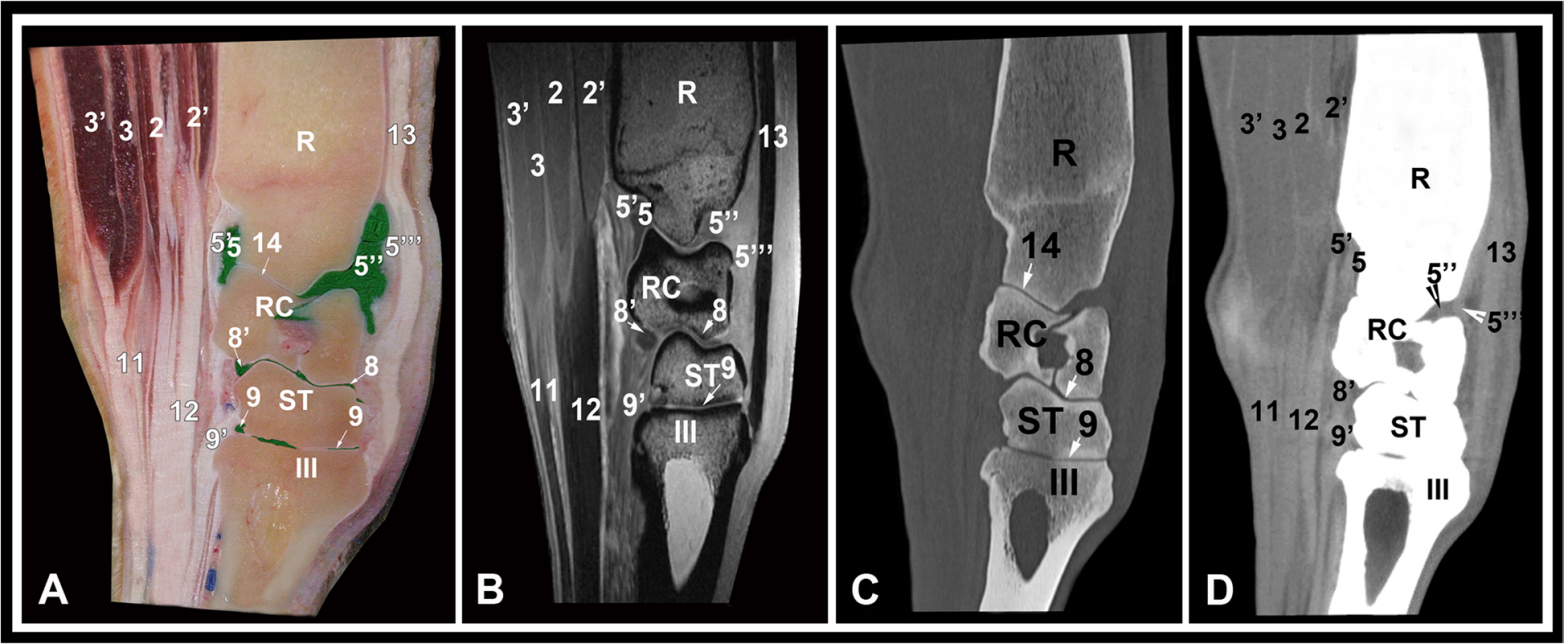
Medial parasagittal gross anatomic (**A**), MRI (**B**), CT bone window (**C**) and CT soft tissue window (**D**) images at the level of the radial carpal bone. R, radius; RC, radial carpal bone; ST, fused 2nd and 3rd carpal bone; III, third metacarpal bone; 2, ulnar and humeral heads of the deep digital flexor muscle; 2, ulnar and humeral heads of the deep digital flexor muscle; 2’, radial head of the deep digital flexor muscle; 3, superficial digital flexor muscle, deep part; 3’, superficial digital flexor muscle, superficial part; 5, palmar recess of the antebrachiocarpal joint; 5’,palmar joint capsule; 5’’, dorsal recess of the antebrachiocarpal joint; 5’’’, dorsal joint capsule; 8, middle intercarpal joint; 8’, palmar recess of the middle intercarpal joint; 9, carpometacatpal joint; 9, carpometacatpal joint, palmar recess; 11, superficial digital flexor tendon; 12, deep digital flexor tendon; 13, extensor carpi radialis tendon; 14, antebrachiocarpal joint

**Fig. 4:**
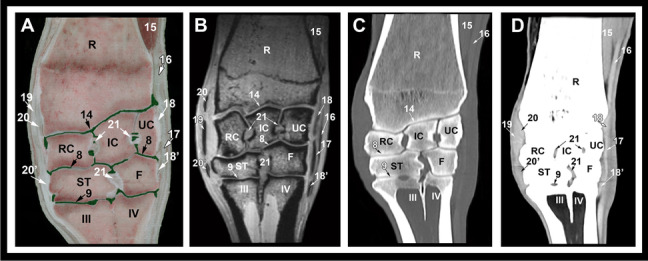
Dorsal gross anatomic (**A**), MRI (**B**), CT bone window (**C**) and CT soft tissue window (**D**) images at the level of the collateral ligaments. R, radius; UC, ulnar carpal bone; IC, intermediate carpal bone; RC, radial carpal bone; ST, fused 2nd and 3rd carpal bone; F, fourth carpal bone; III, 3rd metacarpal bone; VI, fourth metacarpal bone; 8, middle intercarpal joint; 9, carpometacatpal joint; 14, antebrachiocarpal joint; 15, extensor carpi obliquus muscle; 16, common digital extensor tendon; 17, long lateral collateral carpal ligament; 18, short lateral collateral carpal ligament, proximal part; 18’, short lateral collateral carpal ligament, distal part; 19, long medial collateral carpal ligament; 20, short medial collateral carpal ligament, proximal part; 20’, short medial collateral carpal ligament, distal part; 21, short intercarpal ligaments

**Fig. 5:**
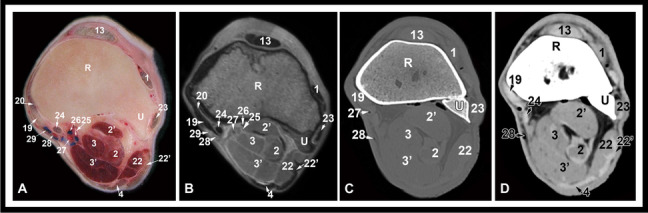
Transverse gross anatomic (**A**), MRI (**B**), CT bone window (**C**) and CT soft tissue window (**D**) images at the level of the distal radius. R, radius; U, ulna; 1, common digital extensor tendon; 2, ulnar and humeral heads of the deep digital flexor muscle; 2’, radial head of the deep digital flexor muscle; 3, superficial digital flexor muscle, deep part; 3’, superficial digital flexor muscle, superficial part; 4, flexor carpi ulnaris tendon; 13, extensor carpi radialis tendon; 19, long medial collateral carpal ligament; 20, short medial collateral carpal ligament, proximal limb; 22, extensor carpi ulnaris muscle; 22’, extensor carpi ulnaris tendon; 23, lateral digital extensor tendon; 24, flexor carpi radialis tendon; 25, median nerve; 26, median vein; 27, medial artery; 28, radial artery; 29, radial vein

**Fig. 6:**
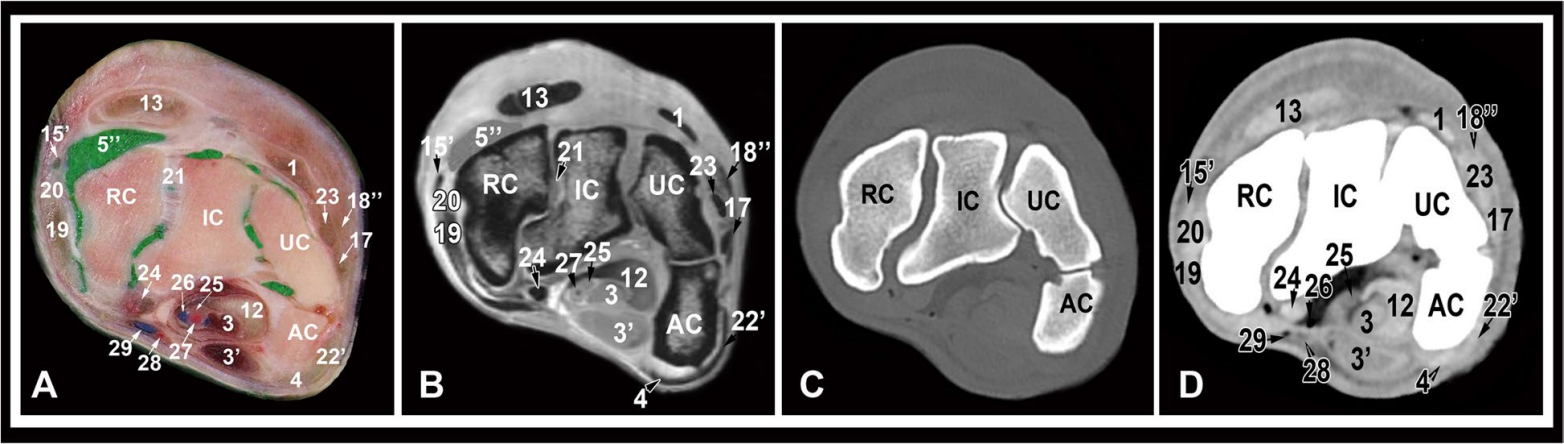
Transverse gross anatomic (**A**), MRI (**B**), CT bone window (**C**) and CT soft tissue window (**D**) images at the level of the proximal row of carpal bones. UC, ulnar carpal bone; IC, intermediate carpal bone; RC, radial carpal bone; AC, accessory carpal bone; 1, common digital extensor tendon; 3, superficial digital flexor muscle, deep part; 3’, superficial digital flexor muscle, superficial part; 4, flexor carpi ulnaris tendon; 5’’, dorsal recess of the antebrachiocarpal joint; 12, deep digital flexor tendon; 13, extensor carpi radialis tendon; 15’ extensor carpi obliquus tedon; 17, long lateral collateral carpal ligament; 18’’, short lateral collateral carpal ligament, middle part; 19, long medial collateral carpal ligament; 20, short medial collateral carpal ligament, proximal limb; 21, short intercarpal ligaments; 22’, extensor carpi ulnaris tendon; 23, lateral digital extensor tendon; 24, flexor carpi radialis tendon; 25, median nerve; 26, median vein; 27, medial artery; 28, radial artery; 29, radial vein

**Fig. 7:**
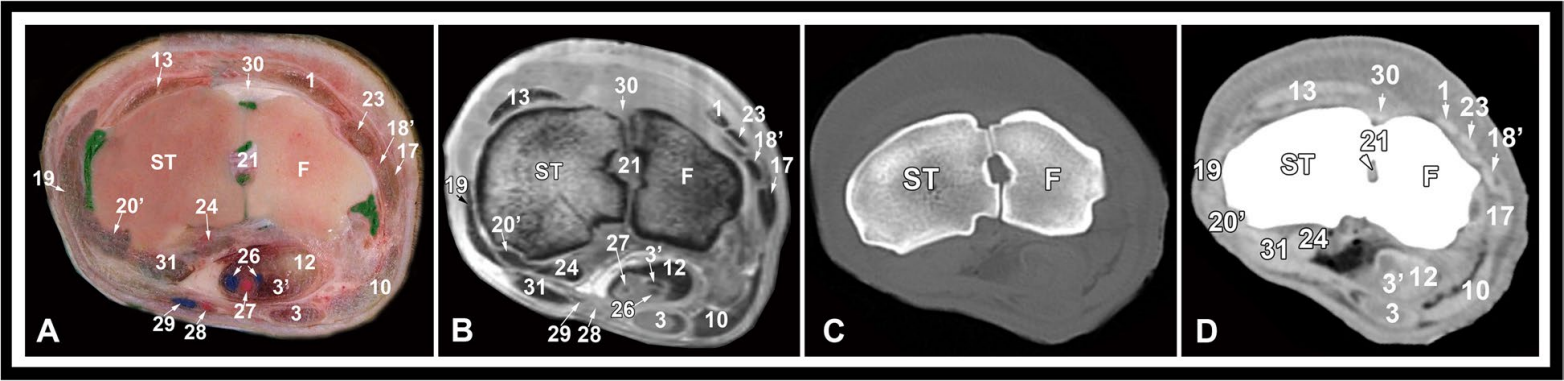
Transverse gross anatomic (**A**), MRI (**B**), CT bone window (**C**) and CT soft tissue window (**D**) images at the level of the distal row of carpal bones. ST, fused 2nd and 3rd carpal bone; F, fourth carpal bone; 1, common digital extensor tendon; 3, superficial digital flexor muscle, deep part; 3’, superficial digital flexor muscle, superficial part; 10, accessoriometacarpal ligament; 12, deep digital flexor tendon; 13, extensor carpi radialis tendon; 17, long lateral collateral carpal ligament; 18’, short lateral collateral carpal ligament, distal limb; 19, long medial collateral carpal ligament; 20’, short medial collateral carpal ligament, distal part; 21, short intercarpal ligaments; 23, lateral digital extensor tendon; 24, flexor carpi radialis tendon; 26, median vein; 27, medial artery; 28, radial artery; 29, radial vein; 30, dorsal intercarpal ligament; 31, reinforcing palmar fibers of the medial collateral ligament

**Fig. 8:**
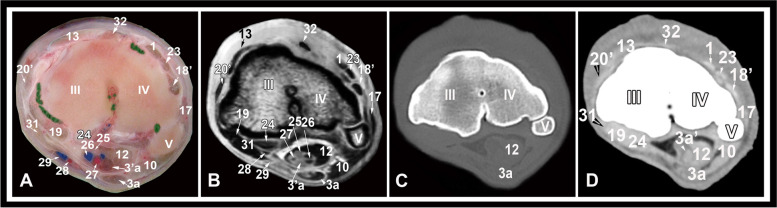
Transverse gross anatomic (**A**), MRI (**B**), CT bone window (**C**) and CT soft tissue window (**D**) images at the level of the proximal metacarpal region. III, third metacarpal bone; VI, fourth metacarpal bone; V, fifth metacarpal bone; 1, common digital extensor tendon; 3a, superficial digital flexor tendon, deep part; 3’a, superficial digital flexor tendon, superficial part; 10, accessoriometacarpal ligament; 12, deep digital flexor tendon; 13, extensor carpi radialis tendon; 17, long lateral collateral carpal ligament; 18’, short lateral collateral carpal ligament, distal limb; 19, long medial collateral carpal ligament; 20’, short medial collateral carpal ligament, distal part; 23, lateral digital extensor tendon; 24, flexor carpi radialis tendon; 25, median nerve; 26, median vein; 27, medial artery; 28, radial artery; 29, radial vein; 31, reinforcing palmar fibers of the medial collateral ligament; 32, dorsal carpometacarpal ligament

The MRI and CT images from 7 sections in 3 planes were selected and adjoined to their corresponding anatomic slices: 2 in the sagittal plane, 1 in the dorsal plane and 4 in the transverse plane (Fig. 1). For each anatomic section, a corresponding MRI and CT images were selected on the basis of similarity. The clinically osseous and soft tissue structures of the bovine carpus were identified and labeled on the anatomic slice then subsequently located on the corresponding CT and MRI images. Each figure is a composite of four images corresponding to the adjoined gross anatomic section, MRI, CT bone window and CT soft tissue window. The transverse and dorsal images were oriented with lateral to the right and sagittal images were oriented with dorsal to the right. The representative sagittal images were selected laterally at the level of the ulnar carpal bone (Fig. 2) and medially at the level of the radial carpal bone (Fig. 3). A dorsal image was chosen at the level of the collateral ligaments (Fig. 4). The transverse images were selected at the level of the distal radius (Fig. 5), proximal row of carpal bones (Fig. 6), distal row of carpal bones (Fig. 7), and the proximal metacarpal region (Fig. 8).

Osseous structures including the distal portion of radius and ulna, contours of the radial trochlea, the proximal and distal rows of carpal bones (radial, intermediate, ulnar, accessory, fused second and third and the fourth carpal bones) and the third, fourth and fifth metacarpal bones could be evaluated on the transverse, sagittal and dorsal CT and MRI images. By the use of CT bone window settings, the cortex and medulla were clearly depicted. The cortical, subchondral and cancellous bone were clearly discriminated and the trabecular pattern of the cancellous bone was well recognized. On the MRI images, the cortex had low signal intensity and medulla showed heterogeneous high signal intensity. The articular cartilage was best evaluated in the sagittal and dorsal images and had intermediate to high signal intensity (Figs. 2, 3 and 4).

The soft tissue structures that could be identified and evaluated on the CT soft tissue window and MRI images included the ulnar, radial and humeral heads of the deep digital flexor muscle; the superficial and deep divisions of the superficial digital flexor muscle; the extensor carpi obliquus muscle; the carpal flexor and extensor tendons (common digital extensor, extensor carpi radialis, lateral digital extensor, superficial and deep digital flexor, flexor carpi radialis and flexor and extensor carpi ulnaris tendons); the long and short limbs of the medial and lateral collateral ligaments; the intercarpal ligaments; the accessoriocarpoulnar, accessorioquartal and accessoriometacarpal ligaments; joint pouches; and the median and radial vessels.

The extensor carpi radialis tendon was best evaluated in sagittal images along the dorsal aspect of the carpus where it terminated on the third metacarpal bone (Figs. 2 and 3). The common digital extensor tendon was identified lateral to the extensor carpi radialis tendon on the dorsolateral aspect of the carpus. Both tendons were markedly hypointense with clearly defined margins on the MRI images and had hyperattenuated density on the CT images (Fig. 5). The extensor carpi obliquus muscle and tendon coursed obliquely from the distolateral aspect of the radius to the medial aspect of the third metacarpal bone. It was occasionally seen on the transverse and dorsal images with ill—defined margins and low to intermediate signal intensity. Due to the oblique position of the muscle in relation to the magnet, magic angle artifact was detected. The tendons of the lateral digital extensor, flexor carpi ulnaris, extensor carpi radialis, and flexor carpi radialis muscles were best evaluated in the transverse images and showed uniform low signal intensity on MRI images and had hyperattenuated density on the CT images (Figs. 5, 6, 7 and 8). The ulnar, humeral and radial heads of the deep digital flexor muscle and the superficial and deep parts of the superficial digital flexor muscle could be differentiated at the level of the distal aspect of the radius as hypodense structures on the CT images and had intermediate signal intensity on the MRI images (Fig. 5). At the level of the proximal row of carpal bones, the tendons of the superficial and deep digital flexor muscles contain variable amount of muscle tissue (muscle–tendon transition) and showed heterogeneous signal intensity and tissue density (Fig. 6). At the level of the distal row of carpal bones, the tendons had uniform low signal intensity on MRI images and hyperattenuated density on the CT images (Fig. 7).

The origin of the long collateral ligaments from the ulnar and radial styloid processes could be evaluated and followed from proximal to distal on the dorsal images (Fig. 4). The short deep collateral ligaments were partially visible as they ran in a nearly horizontal (dorsoproximal-palmarodistal) direction. At the level of the carpal bones, the long lateral collateral ligament appeared thicker than its more proximal part and had heterogeneous low signal intensity with clear margins. The medial collateral ligament had also heterogeneous signal intensity, and was larger, more elongated, and extended around the medial side of the fused second and third carpal bone. At the level of the proximal metacarpal region, the lateral collateral ligament was thin and elongated with heterogeneous density and signal intensity on the CT and MRI images, respectively. The medial collateral ligament became thinner more palmar and extended around the mediopalmar side of the third metacarpal bone (Fig. 8).

The accessoriocarpoulnar, accessorioquartal and accessoriometacarpal ligaments were best evaluated on the sagittal images and showed low to intermediate signal intensity. Due to the orientation of the ligaments around the accessory carpal bone in relation to the magnet, magic angle artifact was noted. The intercarpal ligaments were recognized at the level of the associated joint spaces. The articular surfaces and joint spaces of the carpal joints were clearly defined. The median artery and vein were deeply located medial to the deep part of the superficial digital flexor tendon and appeared as circular structures, of medium to low signal intensity. The radial vein was seen running subcutaneously on the palmaro-medial aspect, accompanied laterally by the radial artery caudal to the flexor carpi radialis tendon. The flexor retinaculum extended from the accessory carpal bone to the medial collateral ligament of the carpus and to the proximal palmar aspects of the second and fourth metacarpal bones forming the palmar wall of the carpal canal. It had homogeneous intermediate to low signal intensity and hyperattenuated tissue density. Synovial fluid was observed in the dorsal and palmar joint pouches, between the carpal bones and between the intercarpal ligament fibers with intermediate signal intensity.

## Discussion

As far as we know, this is the first description of the CT and MRI of the normal bovine carpal joint by the use of multi-detector row CT and 3 T high-field MRI scanner. In the present investigation, the normal T1-weighted MRI signal intensity and the CT tissue density of the articular and peri-articular structures of the bovine carpus were described. The complexity of the bovine carpal joint and the possibility of superimposition of bony structures can be overcome by studying the transverse CT and MRI images of the joint. By studying the sagittal and dorsal images, the entire carpal joint surfaces and joint spaces can be evaluated. Previous CT and MRI studies in horses reported that imaging of joints in three planes provides comprehensive assessment of the clinically significant anatomic structures [14–18]. The images provided in the present study should augment the clinical use of CT for the diagnosis of pathological conditions within the carpal joint that result in clinical lameness.

In the present investigation, the osseous and soft tissue structures identified on the gross anatomic slices correlated well with the CT and MRI images. CT provided excellent delineation of the subchondral and cortical bone, outlined the trabeculae of the cancellous bone and allowed identification of the majority of the clinically relevant soft tissue structures. MRI images provided more details and better definition of the soft tissue structures. The most recognizable difference between CT and MR images was that tendons and ligaments were defined in greater soft tissue contrast in the MRI images and tissue texture was better demonstrated using CT [14]. In this study, the high quality of the CT images were attributed to the use of a multi-row detector spiral CT. Improvements were appreciated to the thinner collimation, quick scanning, flexibility in acquisition and reconstruction and the higher spatial resolution [17]. The detailed and better definition of the 3 T high-field MRI images was associated with the improved coil designs that enabled visualization and characterization of the musculoskeletal structures in exquisite detail and high resolution [19]. In the present study, 3 T T1-weighted MRI images were provided. T1—weighted imaging sequence is the best for evaluation of anatomic details of the articular and periarticular structures and allows good identification of articular cartilage due to the contrast between articular cartilage and the adjacent subchondral bone [9]. On the other hand, T2-weighted images provide better visualization of synovium than T1-weighted images, but resolution and image details are less than optimal for an anatomic study [15, 16]. In horses, CT and T1-weighted MRI images were used to describe the normal CT and MRI anatomy of the equine carpus [14].

The bovine carpus is supported by numerous ligaments and several fibrous bands including the medial and lateral collateral ligaments extending between the forearm and the metacarpus and the short ligaments joining the carpal bones. The collateral ligaments are medial and lateral, uniting all the three joints constituting the carpus [1, 2]. In the previous CT studies of the carpal and fetlock joints in horses, discrimination between the superficial and deep parts of the collateral ligaments was impossible [14, 20] and in the tarsal joint, the subdivisions of the deep collateral ligaments were not visible [21]. In horses the bands of the deep collateral carpal ligaments were occasionally visible with the high-field MRI (1.5 Tesla) and with the low-field MRI (0.27 Tesla) and differentiation between the superficial and deep collateral ligaments was difficult [15, 16, 22]. As performed in the present study, CT and high-field 3 Tesla MRI allowed evaluation and differentiation between the long and short subdivisions of the collateral ligaments and provided thorough evaluation of the ligaments throughout the joint.

Lameness originating from the carpus in cattle may adversely affect the dairy production. Prompt diagnosis and treatment are required to preserve function and produce a desirable outcome; however, detection remains a challenge because cows are good at disguising discomfort [23]. Once lameness has been localized to the carpus, further evaluation is required using radiography and/or ultrasonography. The complexity of the carpal region and myriad of potential sources of pain can make clinically isolating and managing the cause of lameness challenging [16]. The introduction of cross-sectional imaging modalities, specifically CT and MRI has greatly expanded the ability of the radiologist to make these diagnoses [24]. The use of CT and MRI in bovine orthopedics is limited by cost, availability, and the need for general anesthesia. Despite its cost, the use of CT and MRI should be considered as a diagnostic tests and decision-making tools for the economically valuable cattle. Delayed diagnosis may lead to economic loss. Therefore, the cost savings per animal may well be beneficial if early diagnosis is achieved through the use of such modalities. Furthermore, repeated treatments without correct diagnosis can lead to great economic losses [25]. Reports demonstrating the clinical utility of these technologies arise with increasing frequency in the veterinary literature. In cattle, CT was successfully used for diagnosis of neurological diseases, skeletal disorders, metastatic tumors, sinusitis, actinomycosis, bronchopneumonia, tympanosis, and morphological changes in the eyes and thyroid glands [11, 25–30]. In horse, CT has proven to detect subchondral and occult osteochondral lesions [24] and to evaluate complex comminuted fractures when radiographic interpretation is difficult thanks to the cross-sectional images with spatial separation of the superimposed structures seen on survey radiographs. This allows accurate assessment of the number and direction of the fracture lines within the bone [31]. MRI has high sensitivity and specificity for detecting soft tissue lesions in the absence of positive findings with other imaging modalities [15]. It is possible to acquire MRI images of the carpus in either low or high-field MRI systems. High-field images provide more details and knowledge of high-field anatomy is helpful for interpretation of low-field MRI images [16]. MRI showed great development that led to improved gradient performance and broadened the clinical applications for the 3 T MRI imaging systems [12]. The 3 T systems are characterized by superior coil design and better gradient performance, compared with the 1.5 T MRI systems and provides better contrast, improve spatial resolution and shorten acquisition times as well as decreased echo spacing to lessen blurring and image distortion [32]. These capabilities are particularly advantageous for evaluating of complex joints (such as the carpus) that includes many small but clinically important soft-tissue structures (ligaments, fibrocartilage, hyaline cartilage, and regional nerves) that are often difficult to differentiate due to their limited inherent contrast [19].

In the present study, high-field MRI provided highly detailed visualization and differentiation of soft tissue structures particularly tiny structures as the subdivisions of the collateral ligaments. CT allowed qualitative description of the osseous and soft tissues of the bovine carpus. The images provided should augment the clinical use of CT and MRI for the diagnosis of pathological conditions within the carpal joint that result in clinical lameness.

## Conclusions

The structures of the bovine carpus are numerous, uniquely orientated relative to each other, and have specific anatomic or structural variations individual to each structure. High-field MRI provided highly detailed assessments of the bovine carpus, particularly soft tissues. CT provided the best bone images and soft tissue structures were also identified on the CT soft tissue window images, but never with the definition provided by the MRI images. Interpretation of the acquired CT and MRI images is a challenge and requires a good knowledge of the normal anatomy and a clear understanding of the CT and MRI physics before diagnoses can be made with confidence. This study provides anatomical information on the bovine carpus, which can be useful in both clinical and research use. This information can serve as a baseline reference for evaluation of the CT and MRI scans of the bovine carpal joint in clinical circumstances.

## Methods

### Animals

Twelve normal forelimbs (*n* = 12) were used to acquire the CT and 3 Tesla MRI images. Limbs were collected from 6 adult Holstein Friesian cow cadavers referred to the Institute of Veterinary Pathology, Faculty of Veterinary Medicine, Leipzig University and euthanized for medical reasons unrelated to the study. The age of cows ranged from 5 to 12 years and weight ranged from 450–550 kg. Immediately after euthanasia, limbs were disarticulated at the elbow joint to maintain normal anatomic positioning and scanned within 2 h of euthanasia. The carpal region in each limb was grossly investigated then examined by radiography (0 , 90 , 45 , and 135  views) and ultrasonography to confirm absence of abnormalities.

### MRI protocol

Magnetic resonance scanning was acquired in a high‐field 3 Tesla MRI (Philips Ingenia; Philips AG) scanner using a human extremity radiofrequency coil. The limbs were extended and placed with the lateral aspect as the dependent portion and the long axis of the limb parallel to the MRI table (to mimic the limb of a cow in lateral recumbency). A T1-weighted turbo spin echo (TSE) was used to obtain the best anatomical detail of the soft tissues of the joint in sagittal, dorsal, and transverse planes and the signal intensity of each structure was reported. Images were acquired with the following settings: repetition time, 600 ms; echo time, 19 ms; slice thickness, 3 mm; inter-slice gap, 0.3 mm; flip angle, 90°; field of view, 200 × 98; and reconstructed matrix of 1024 × 1024. Representative images of the clinically relevant anatomic structures of the carpal joint at various levels that were best correlated with the gross anatomic slices were selected.

### CT scanning

Following MRI, CT scanning of the carpal joint was acquired using a multi-detector 16-slice helical CT scanner (Philips Mx8000 IDT 16-slice helical CT scanner; Philips, GmbH, Hamburg, Germany). The limbs were extended and placed on the CT table as mentioned in the MRI study. A survey image (120 kV, 80 mA) was performed to check for symmetry and to ensure that the entire region of interest was included. Acquisition variables were: 120 kV, 80 mA, slice thickness of 3 mm, inter-slice space of 1 mm, rotation time of 1 s, pitch of 0.63, field of view 20 cm, and matrix size of 512 X 512. The transverse images were reconstructed into sagittal, and dorsal planes and reviewed by the use of a bone setting (window width, 2700 HUs; window level, 350 HUs) and a soft tissue setting (window width, 320 HUs; window level, 30 HUs). Each CT image volume was used to generate a three-dimensional representation of the carpal joints. The opacity of all structures was observed, noted and matched to the corresponding anatomic sections. Features in the CT images that corresponded to the clinically relevant anatomic structures in tissue sections were identified.

### Anatomic specimen preparation

At the conclusion of the MRI and CT examinations, the arteries, veins, and synovial structures in the carpal region (same limbs that underwent CT and MRI examinations) were injected with red, blue, and green latex, respectively. The injection needle was inserted between the extensor carpi radialis and common digital extensor tendons. In the antebrachiocarpal joint, the needle was inserted between the distal aspect of radius and the dorsal rim of intermediate carpal bone and between the proximal and distal rows of carpal bones in the middle carpal joint. The carpometacarpal joint communicates with the middle carpal joint and, therefore, it did not require a separate entry for injection. Synovial fluid was aspirated and replaced with the green latex. Arterial and venous injections were carried out via the brachial artery and vein. Limbs were cooled at 4 °C for two days until latex became hard, and then frozen at – 18 °C for two weeks. The frozen limbs were transected in 5-mm-thick slices in sagittal, dorsal, or transverse planes from the mid of the radius to the proximal metacarpus using an electric band saw. The cut surfaces of each section were cleaned and the front and back faces of the anatomic slices were photographed.

## Data Availability

The datasets used and/or analyzed during the current study are available from the corresponding author on reasonable request.

## Abbreviations

MRI: Magnetic resonance imaging
CT: Computed tomography
TSE: Turbo Spin-echo; T1-weighted
